# Water-level fluctuations and metapopulation dynamics as drivers of genetic diversity in populations of three Tanganyikan cichlid fish species

**DOI:** 10.1111/mec.12374

**Published:** 2013-07-10

**Authors:** B Nevado, S Mautner, C Sturmbauer, E Verheyen

**Affiliations:** * Centre for Research in Agricultural Genomics, Universitat Autònonoma de Barcelona08193, Bellaterra, Spain; † Department Vertebrates, Royal Belgian Institute of Natural SciencesVautierstraat 29, 1000, Brussels, Belgium; ‡ Department of Zoology, Karl-Franzens-University GrazUniversitätsplatz 2, 8010, Graz, Austria; § Evolutionary Ecology Group, University of AntwerpGroenenborgerlaan 171, 2020, Antwerp, Belgium

**Keywords:** genetic diversity, lake-level fluctuations, metapopulation dynamics, Tanganyikan cichlids

## Abstract

Understanding how genetic variation is generated and maintained in natural populations, and how this process unfolds in a changing environment, remains a central issue in biological research. In this work, we analysed patterns of genetic diversity from several populations of three cichlid species from Lake Tanganyika in parallel, using the mitochondrial DNA control region. We sampled populations inhabiting the littoral rocky habitats in both very deep and very shallow areas of the lake. We hypothesized that the former would constitute relatively older, more stable and genetically more diverse populations, because they should have been less severely affected by the well-documented episodes of dramatic water-level fluctuations. In agreement with our predictions, populations of all three species sampled in very shallow shorelines showed traces of stronger population growth than populations of the same species inhabiting deep shorelines. However, contrary to our working hypothesis, we found a significant trend towards increased genetic diversity in the younger, demographically less stable populations inhabiting shallow areas, in comparison with the older and more stable populations inhabiting the deep shorelines. We interpret this finding as the result of the establishment of metapopulation dynamics in the former shorelines, by the frequent perturbation and reshuffling of individuals between populations due to the lake-level fluctuations. The repeated succession of periods of allopatric separation and secondary contact is likely to have further increased the rapid pace of speciation in lacustrine cichlids.

## Introduction

The role of small- vs. large-scale environmental changes in the generation and maintenance of genetic variation in natural populations remains a central but neglected issue in biological research (Leffler *et al*. 2012). Levels of genetic diversity within populations will depend on the net balance between gain and loss of genetic variants, but while mechanisms behind the generation of new genetic variants are relatively uncontroversial, those involved in their maintenance or disappearance remain the subject of debate. The neutral theory of molecular evolution (Kimura 1983) forms the most widely accepted null hypothesis in evolutionary genetics and comparative genomics posing that most evolution at the molecular level is driven by mutation and random drift (the loss of genetic variation due to random sampling of gametes in finite populations, Kimura & Crow 1964), rather than selection. Under this scenario, species with larger and more stable population sizes are expected to maintain higher levels of neutral genetic diversity due to the reduced effect of genetic drift, while more complex interactions are expected for genetic variation under direct or indirect (e.g. via linkage) selection (Smith & Haigh 1974; Charlesworth *et al*. 1993). While a central and relatively simple prediction, this correlation between population sizes and stability on one hand, and genetic diversity levels on the other, remains divisive (e.g. Bazin *et al*. 2006; Crow 2008; Leffler *et al*. 2012). In this work, we test the hypothesis that neutral genetic diversity levels correlate with population stability in natural populations of cichlid fish from Lake Tanganyika.

**Fig. 1:**
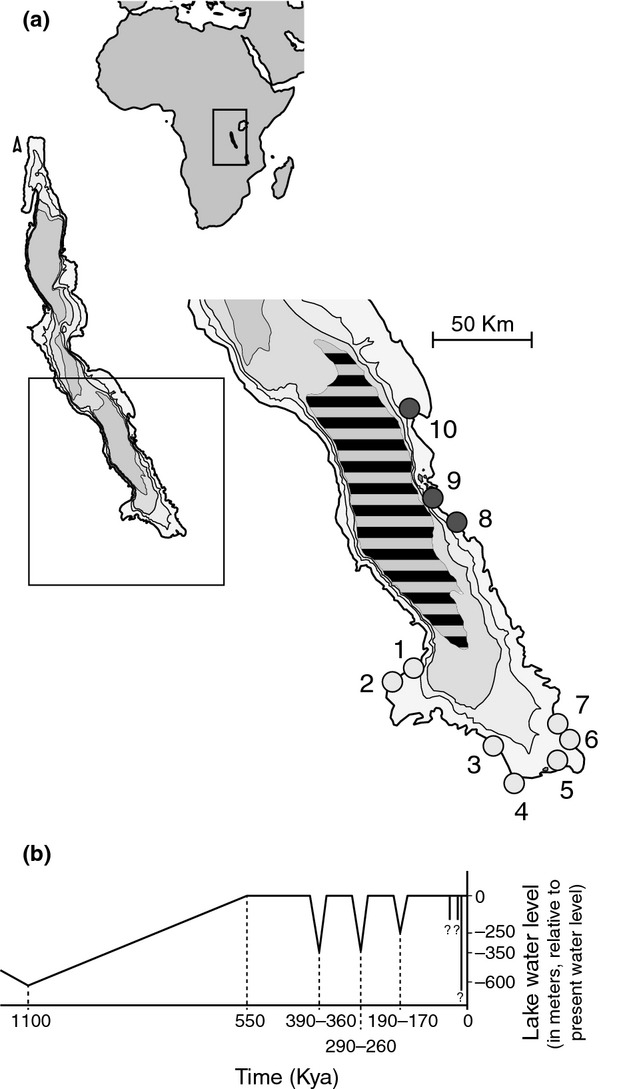
a) Map detailing the 10 localities sampled in this study. Inset at top shows location of Lake Tanganyika in East Africa. Localities are numbered 1–10 according to Table 1. Localities 8–10 were classified as deep and are represented with a darker shade, and remaining localities were classified as shallow and are marked with a lighter shade. Thin lines inside the lake are bathymetric lines of 250 and 500 m below present lake level. Dashed area marks approximate shoreline location following a drop of c. 600 m below present level. b) Reconstruction of lake-level low stands based on Lezzar *et al*. (1996) and Cohen *et al*. (1997). The vertical lines on the right indicate the most recent lake-level low stands, question marks denote that the exact magnitude of these changes is uncertain (adapted from Baric et al. 2003).

Lake Tanganyika is the second oldest lake in the world, well known for harbouring the ecologically, morphologically and behaviourally most diverse cichlid species flock in the world (Fryer & Iles 1972; Poll 1986), totalling an estimated number of 250 endemic species (Turner *et al*. 2001). Geological evidence suggests that the lake started to form about 9 to 12 Mya (million years ago) and that at that time, Lake Tanganyika consisted of at least three shallow, swampy proto-lakes (Cohen *et al*. 1997). Tectonic activity deepened these basins until they fused to a single deep clearwater lake around 5–6 Mya (Tiercelin & Mondeguer 1991; Cohen *et al*. 1993). A change towards a drier climate in the late Pliocene to early Pleistocene led to a major water-level low stand (650–700 m below present level) about 1.1 Mya. Following this major decrease, the water level rose again attaining its present level c. 550 Kya (thousand years ago; Lezzar *et al*. 1996; Cohen *et al*. 1997). Paleoclimate and geological records show that new drops in the lake level occurred 390–360, 290–260 and 190–170 Kya reaching down to 250–350 m below present level (Cohen *et al*. 1997). Moreover, the lake level was also substantially lower on several occasions during the late Pleistocene glacial cycles, when the climate in north and equatorial Africa became progressively more arid. Precise timing and extent of these changes remains contentious (Gasse *et al*. 1989; Lezzar *et al*. 1996; Cohen *et al*. 1997); however, most studies suggest that these major drops in water level occurred between 135 and 60 Kya, with drops of up to 500–600 m below present level (Cohen *et al*. 2007; Scholz *et al*. 2007). Given the structure of Lake Tanganyika's basin, a drop of c. 600 m below present water level should result in two or three isolated sub-basins within the lake's catchment (Fig. 1).

Because changes of the lake level affect shoreline structure and connectivity patterns, they have long been proposed as drivers of population subdivision and secondary contact cycles (Fryer 1959; Sturmbauer & Meyer 1992; Verheyen *et al*. 1996; Sturmbauer 1998; Sturmbauer *et al*. 2001; Egger *et al*. 2007), in a process termed ‘species pump’ (Rossiter 1995). Given that most cichlid fish species inhabiting the East African Great Lakes are littoral species with very specific habitat requirements and relatively low dispersal ability, the changes to connectivity of habitat usually leave clear signatures in the genetic variation of populations and species. Furthermore, the lake's varied bathymetric profile suggests that water-level fluctuations can have very different effects upon fish populations inhabiting different shoreline sections. In the middle sections of the three sub-basins, the shoreline drops almost uninterruptedly to c. 1400 m below present levels, while at the southern and northern edges of the lake, the inclination is much weaker, resulting in these regions being shallower and emergent during periods of low water levels (Fig. 1). Thus, populations inhabiting the former localities could be regarded as relatively stable, being close to a deep, permanent lake basin, while those inhabiting the latter shoreline sections are likely to be comparatively young, and have experienced more dramatic changes to their population sizes and habitat availability.

The hypothesis that shorelines at deeper basins harbour relatively older and more stable populations has recently been tested using populations of the Lake Tanganyika cichlid fish species *Tropheus moorii* from the southern end of the lake (Koblmüller *et al*. 2011). In accordance with expectations, the authors estimated more pronounced demographic changes and younger ages for populations inhabiting shallower areas, whereas those at deeper basins generally harboured older and demographically more stable populations of *T. moorii*. More specifically, the analysis by Koblmüller *et al*. (2011) found a strong influence of late Pleistocene water-level fluctuations (up to 50–100 Kya) on the populations inhabiting the southern edge of Lake Tanganyika, in accordance with recent results for Lake Malawi's cichlid fauna (Genner *et al*. 2010). However, two questions remain unclear: (i) whether the relationship between shoreline depth and population genetic diversity is a general pattern across cichlid species; and (ii) whether this relationship extends to longer timescales than those considered by the previous study's authors.

We aimed to answer these two questions by comparing the genetic diversity of fish populations sampled from different localities in both the southernmost end of Lake Tanganyika, which is emergent during periods of low water level, and in the deep middle section of the southern sub-basin (Fig. 1). We analysed the control region of the mitochondrial DNA (mtDNA) of populations of three littoral Tanganyikan cichlid species, *Variabilichromis moorii*, *Tropheus moorii* and *Eretmodus cyanostictus*, assigned to the tribes Lamprologini, Tropheini and Eretmodini (Poll 1986). The study species are often found living in sympatry, and share several ecological and life history characteristics. They all have a preference for rocky shallow habitats, a mostly herbivorous diet, they show territoriality towards conspecifics and lack sexual dimorphism (Kohda *et al*. 1997; Yamaoka *et al*. 1997; Yuma & Kondo 1997). Importantly, all three species are stenotopic rock dwellers with restricted dispersal ability, particularly across nonrocky substrate (Duftner *et al*. 2006; Sefc *et al*. 2007), and thus, their demographic and evolutionary histories are likely to be strongly affected by water-level fluctuations. Furthermore, populations of all three species occur in both the shallow southern end of the lake and in the deep shorelines of the central region of the southern sub-basin. As such, the comparative analysis of populations from these two regions should allow us to test the hypothesis that the latter populations are more stable than those inhabiting the former shoreline sections, and gain insight into mechanisms determining genetic diversity over longer time frames than those investigated by Koblmüller *et al*. (2011).

## Materials and methods

The three target species, *Eretmodus cyanostictus*, *Variabilichromis moorii and Tropheus moorii*, were collected by gillnetting in different localities throughout the lake during different expeditions to Lake Tanganyika (Table 1; Table S1, Supporting Information). Within each locality, sampling was performed within 50–100 m of continuous rocky shoreline and specimens were identified by EV and CS, who were present during all expeditions. Localities sampled included the very deep shorelines situated at the east coast of the southern sub-basin of the lake, as well as the shallow regions in the southern end of Lake Tanganyika (Fig. 1). It must be noted that *T. moorii*'s taxonomic status is at present uncertain, with over 100 geographical colour morphs described from different shorelines in Lake Tanganyika (Schupke 2003; Sturmbauer *et al*. 2005). As such, for the remainder of this work, we refer to populations of this species as *Tropheus* sp.

**Table 1:** Number of individuals of each species collected across localities sampled (localities’ numbers according to Fig. 1)

*Locality*	*E. cyanostictus*	*V. moorii*	*Tropheus* sp.
1	38	41	55 (35)*
2	11	42	46
3	40	46	26
4	38	41	50 (23/19)*
5	28	35	46 (42)*
6	—	24	46
7	29	37	—
8	17	50	40 (19/21)*
9	37	48	48
10	—	12	25 (15)*
Total	238	376	382

* In some localities, more than one mtDNA lineage was re-covered in samples of *Tropheus* sp. Values inside parenthesis denote the number of individuals belonging to the most abundant mtDNA lineage(s).

For all specimens collected, fin or muscle tissue was preserved in 80% ethanol for subsequent molecular analysis. DNA was extracted using standard protocols, and amplification and sequencing of the first most variable part of the mtDNA control region was performed according to protocols specified in Table S2 (Supporting Information).

DNA sequences obtained were aligned with the program clustalw (Larkin *et al*. 2007) for each species separately, and resulting data sets checked by eye using the program seaview (Gouy *et al*. 2010). Aligned data sets are available from Dryad doi:10.5061/dryad.m2661.

For each species, population differentiation between localities within each species was estimated with Fst (Hudson *et al*. 1992) using the software dnasp v 5.10 (Librado & Rozas 2009).

For each locality of each species, we estimated standard diversity indices (number of segregating sites, number of haplotypes, haplotype diversity, nucleotide diversity and theta) and the following neutrality tests: Tajima's D (Tajima 1989), Fu and Li's D and F (Fu & Li 1993), Fu's Fs (Fu 1997) and Ramos-Onsins R2 (Ramos-Onsins & Rozas 2002). The program dnasp was used to estimate these statistics. Significance of departures from neutrality was calculated with coalescent simulations (1000 replicates used). Mismatch distributions and haplotype networks for each locality of each species were also plotted (using dnasp for the former and tcs, Clement *et al*. 2000, for the latter). Mismatch distributions and haplotype networks both reflect the relationship between haplotypes present in the population under analysis and can be used to infer the demographic history of the population. For instance, strong population growth usually results in bell-shaped mismatch distributions, while relatively constant population sizes lead to multimodal mismatch distributions. As for haplotype networks, population growth usually results in a single abundant haplotype, and many closely related but less abundant haplotypes, while population decreases or substructuring often leads to the disappearance of intermediate haplotypes and longer branches connecting the re-covered haplotypes.

To gauge the effect of inhabiting the different shoreline sections, we classified the localities sampled as either shallow or deep according to their position in relation to the lake's sub-basins (Fig. 1) and performed two statistical tests comparing patterns of genetic diversity between these two locality types: one within each species and a second across all species. For the former, we applied Wilcoxon–Mann–Whitney (WMW) tests (e.g. Hollander & Wolfe 1999) to test for significant differences in genetic diversity indices (haplotype diversity, nucleotide diversity and theta) between populations sampled in the shallow vs. those sampled in the deep localities within each species. For the latter, we performed a two-way analysis of variance (anova) taking the same three genetic diversity indices as the response variables, and the species, locality type and interaction between the two as independent effects. For this analysis, we used data from individual lineages within *Tropheus* sp. when more than one mitochondrial lineage was present in the same locality. Homogeneity of variances in haplotype diversity, nucleotide diversity and theta values across species was confirmed with Bartlett's test (Bartlett 1937) before performing anova. All statistical tests were performed using the software r (http://www.r-project.org).

The program beast v 1.5.3 (Drummond & Rambaut 2007) was used to reconstruct past demographic histories using individual sequences from each locality of each species. Parameters of the best nucleotide substitution model (as selected by jmodeltest, Posada 2008) were estimated in beast (except for the nucleotide frequencies, for which empirical values were used). We implemented a strict molecular clock, and priors for population size were obtained using the Bayesian Skyline method (Drummond *et al*. 2005) with 10 groups. Sampling was set to once every 1000 steps for a minimum of 10 million steps and a maximum of 100 million steps (depending on data sets) to achieve effective sample sizes (ESS) over 200. We checked for convergence of independent runs using tracer by plotting the change in likelihood values through each run and by comparing results of two independent runs. As the different runs achieved similar results, we combined the output of two runs (using logcombiner, part of the Beast package) and plotted the estimated population size changes through time. To provide an approximate time frame for the demographic histories re-covered, we employed a substitution rate of 0.0325–0.057 per site per million of years (Sturmbauer *et al*. 2001; Koblmüller *et al*. 2009).

## Results

Population differentiation within each species was generally high (Table 2) with Fst values above 0.5 for most pairwise comparisons of localities. Among the 238 sequences of *E. cyanostictus,* we recovered 95 unique haplotypes, out of which only 7 were shared across localities. For *V. moorii*, we found 97 haplotypes (376 sequences) with 4 haplotypes present in more than 1 locality. Haplotype diversity within *Tropheus* sp. was higher, with 176 haplotypes recovered from 382 sequences analysed, but only 5 of these haplotypes were found in more than 1 locality. No haplotypes were found in more than 2 (usually neighbouring) localities.

**Table 2:** Estimated Fst values between populations within each species

Locality	*E. cyanostictus*									
1										
2	0.028									
3	0.584	0.546								
4	0.685	0.636	0.133							
5	0.667	0.618	0.211	0.238						
7	0.775	0.725	0.336	0.275	0.336					
8	0.866	0.809	0.758	0.854	0.860	0.919				
9	0.729	0.659	0.673	0.771	0.786	0.846	0.747			

	*V. moorii*									
1										
2	0.209									
3	0.690	0.702								
4	0.613	0.628	0.441							
5	0.358	0.432	0.428	0.364						
6	0.537	0.569	0.746	0.655	0.349					
7	0.526	0.572	0.693	0.586	0.292	0.428				
8	0.557	0.512	0.749	0.662	0.486	0.617	0.628			
9	0.537	0.532	0.715	0.644	0.467	0.524	0.543	0.401		
10	0.828	0.847	0.895	0.826	0.740	0.841	0.857	0.874	0.675	

	*Tropheus* sp. S *									
1										
2	0.416									
3	0.696	0.839								
4	0.513	0.637	0.346							
5	0.674	0.805	0.729	0.317						
6	0.696	0.824	0.742	0.349	0.220					
8	0.559	0.697	0.391	0.155	0.438	0.455				
9	0.345	0.486	0.817	0.601	0.784	0.804	0.659			
10	0.641	0.794	0.697	0.290	0.333	0.370	0.358	0.749		

	*Tropheus* sp. L*									
1 (35)										
2	0.696									
3	0.764	0.830								
4 (19)	0.815	0.886	0.806							
4 (23)	0.820	0.898	0.401	0.889						
5 (42)	0.757	0.826	0.733	0.303	0.812					
6	0.740	0.809	0.699	0.407	0.776	0.262				
8 (19)	0.818	0.897	0.363	0.886	0.535	0.803	0.772			
8 (21)	0.762	0.858	0.763	0.687	0.845	0.580	0.532	0.835		
9	0.601	0.486	0.807	0.866	0.879	0.806	0.785	0.876	0.828	
10 (15)	0.834	0.932	0.830	0.756	0.925	0.573	0.510	0.925	0.534	0.905

* For *Tropheus* sp. we present results per locality (*Tropheus* sp. S) and per lineage (*Tropheus* sp. L, lineages referred to according to Table 1).

**Fig. 2:**
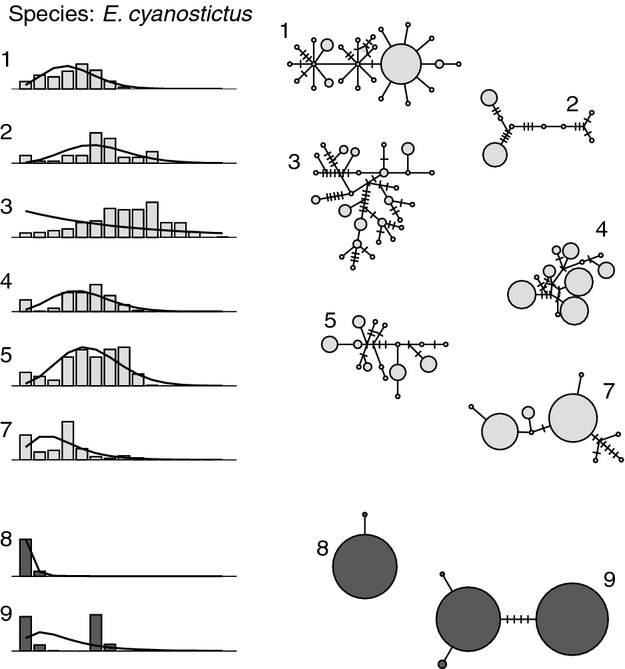
Mismatch distributions (left) and haplotype networks (right) for populations of *E. cyanostictus*. Numbers denote population of origin (according to Table 1). Light shades note populations from shallow areas, darker shades note populations from deep shorelines. Black lines on mismatch distributions denote expected distributions under the model of sudden expansion. In the haplotype networks, each circle represents a different haplotype, with its size reflecting relative abundance. Lines connecting haplotypes represent mutations, and short lines crossing these represent missing haplotypes.

Results of the analysis of genetic variation within populations of each species are depicted in Figs 42 and Table 3. For *E. cyanostictus,* highest values for haplotype diversity, nucleotide diversity and theta were all found in the southern, shallow localities (Table 3). Haplotype networks and mismatch distributions reflect the higher diversity in this area of the lake, with mismatch distributions exhibiting much higher values than the localities at deep shorelines of the southern sub-basin (Fig. 2). Also, many more missing haplotypes were recovered in the localities from the southern shallow shorelines.

**Table 3:** Summary statistics for all populations of the three species analysed: number of sequences (N), segregating sites (S), haplotypes (h), haplotype diversity (Hd), nucleotide diversity (Pi), average number of pairwise differences (k) and theta

Locality	Shoreline*	N	S	h	Hd	Pi	k	θ
*E. cyanostictus*								
1	S	38	25	25	0.929	0.009	3.303	0.017
2	S	11	15	8	0.927	0.016	5.236	0.016
3	S	40	44	27	0.979	0.020	7.000	0.030
4	S	38	15	11	0.888	0.012	4.088	0.010
5	S	28	20	14	0.937	0.013	4.698	0.015
7	S	29	15	9	0.761	0.007	2.601	0.011
8	D	17	1	2	0.118	0.000	0.118	0.001
9	D	37	7	4	0.589	0.008	2.727	0.005
*V. moorii*								
1	S	41	20	19	0.918	0.009	3.066	0.013
2	S	42	17	13	0.873	0.007	2.352	0.011
3	S	46	15	12	0.695	0.006	2.130	0.010
4	S	41	21	13	0.87	0.011	3.867	0.010
5	S	35	22	15	0.83	0.015	5.382	0.015
6	S	24	7	7	0.605	0.006	2.221	0.005
7	S	37	8	6	0.76	0.005	1.931	0.005
8	D	50	10	7	0.327	0.004	1.429	0.006
9	D	48	9	4	0.506	0.010	3.394	0.006
10	D	12	3	2	0.167	0.001	0.500	0.003
*Tropheus* sp.**								
1	S	55	36	28	0.953	0.026	9.206	0.022
1 (35)	S	35	23	19	0.934	0.017	5.945	0.016
2	S	46	17	16	0.91	0.006	2.178	0.011
3	S	26	24	17	0.951	0.012	4.342	0.018
4	S	50	38	19	0.906	0.036	12.960	0.025
4 (23)	S	23	10	7	0.676	0.005	1.708	0.008
4 (19)	S	19	12	8	0.865	0.006	2.164	0.010
5	S	46	41	25	0.961	0.019	6.966	0.026
5 (42)	S	42	31	22	0.955	0.015	5.273	0.020
6	S	46	35	21	0.943	0.018	6.472	0.022
8	D	40	37	19	0.941	0.031	11.050	0.024
8 (21)	D	21	23	12	0.924	0.012	4.190	0.018
8 (19)	D	19	6	7	0.825	0.004	1.368	0.005
9	D	48	12	12	0.839	0.007	2.590	0.008
10	D	25	28	12	0.803	0.021	7.493	0.021
10 (15)	D	15	4	4	0.467	0.002	0.648	0.003

* Localities classified according to shoreline position: Deep (D) or Shallow (S).

** For *Tropheus* sp., we present results per locality and per lineage (lineages referred to according to Table 1).

**Fig. 3:**
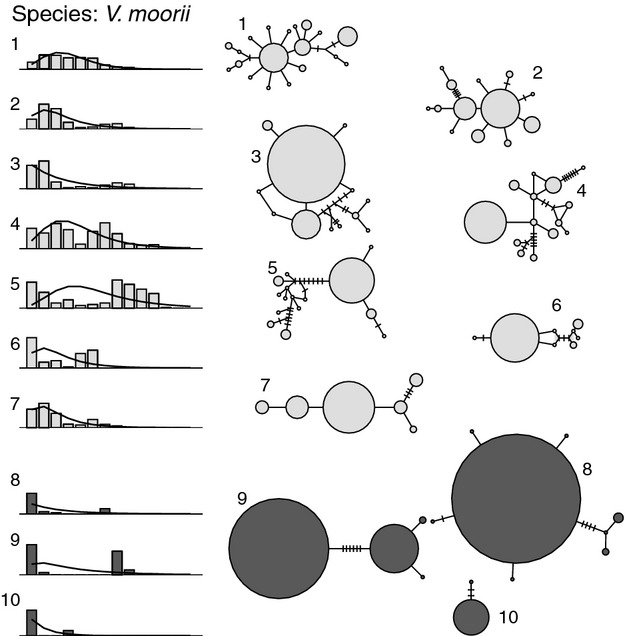
Mismatch distributions (left) and haplotype networks (right) for populations of *V. moorii*. Data presented as in previous figure (see details in legend to Fig. 2).

**Fig. 4:**
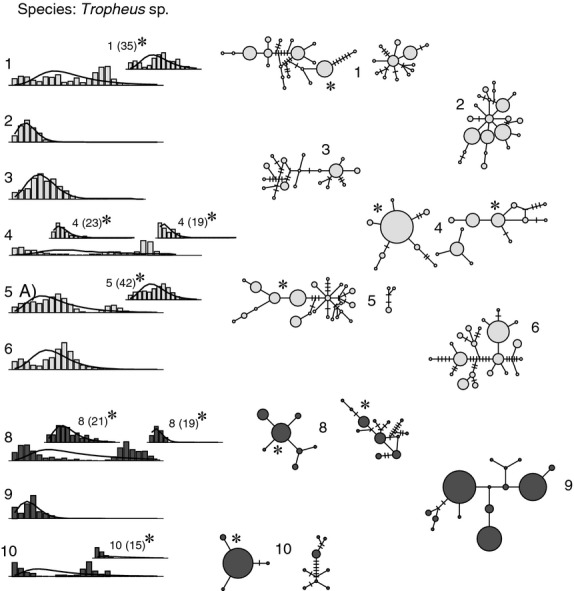
Mismatch distributions (left) and haplotype networks (right) for populations of *Tropheus* sp. Data presented as in previous figures (see details in legend to Fig. 2). As several mtDNA lineages were found in some localities, we show results using all the samples from each locality, and as well using only the samples belonging to the most abundant mtDNA lineage(s) (denoted with * and named according to Table 1).

For *V. moorii* (Fig. 3 and Table 3), a similar pattern was observed: haplotype diversity, nucleotide diversity and estimated theta all pointed to a reduced amount of variation in the deep shorelines around the central part of the southern basin of the lake (localities 8–10). The same pattern became evident in the mismatch distributions and haplotype networks, with localities in the southern, shallow shorelines harbouring a larger number of haplotypes, as well as many more missing haplotypes, in comparison with the localities located in deep shorelines.

For *Tropheus* sp., interpretation of the results regarding genetic variation (Fig. 4 and Table 3) needs to take into account the existence of different mtDNA lineages, some of which already reached reproductive isolation and mate assortatively (Salzburger *et al*. 2006; Egger *et al*. 2008, 2010). Most mtDNA lineages at least reflect different colour morphs that are allopatric; however, in some localities, two or three different mtDNA lineages co-exist. This became also evident on our haplotype networks estimated in tcs, with the members of some populations being resolved in two or more haplotype networks which could not be connected within the 95% parsimony criterion (Templeton *et al*. 1992). Analysis of the networks showed that these different mtDNA lineages correspond to mtDNA clades as defined previously (Baric *et al*. 2003; Sturmbauer *et al*. 2005). Given that genetic diversity indices, mismatch distributions and demographic inferences should all be carried out in panmictic populations, in localities with more than one *Tropheus* sp. mtDNA lineage present, we carried out two analyses: (i) including all the specimens collected in that locality; and (ii) only those specimens belonging to the most abundant mtDNA lineage (or to both mtDNA lineages when the number of individuals belonging to each lineage was roughly equal). While we present all results in Fig. 4 and Tables 2–4, our discussion will focus on the results for single mtDNA lineages, as these are more likely to yield valid estimates of population genetic parameters.

**Table 4:** Result (*P*-values) of the Wilcoxon–Mann–Whitney tests for differences in haplotype diversity (Hd), nucleotide diversity (Pi) and theta estimates between populations classified as Deep (D) or Shallow (S) within each species

	Ha*	Hd	Pi	θ
*E. cyanostictus*	D ≠ S	0.071	0.143	0.071
	D > S	1	0.964	1
	S > D	**0.036**	0.071	**0.036**
*V. moorii*	D ≠ S	**0.0167**	0.2667	0.1833
	D > S	1	0.908	0.942
	S > D	**0.0083**	0.133	0.0917
*Tropheus* sp. S**	D ≠ S	0.095	0.905	0.548
	D > S	0.976	0.452	0.809
	S > D	**0.048**	0.643	0.274
*Tropheus* sp. L**	D ≠ S	0.109	0.164	0.109
	D > S	0.964	0.946	0.964
	S > D	0.055	0.082	0.055

* Alternative hypothesis for the WMW test. D ≠ S is a two-tailed test of difference in mean values. D > S and S > D are one-tailed test of the direction of such differences.

** For *Tropheus* sp., WMW tests were performed using all the individuals in each locality (*Tropheus* sp. S) or only individuals carrying the most abundant mtDNA lineage(s) (*Tropheus* sp. L).

Significant p-values denoted in bold.

Haplotype diversity and theta values in *Tropheus* sp. populations were again lowest at deep shorelines, while higher values were (on average) observed in the shallow localities at the southern end of the lake (Table 3). Nucleotide diversity exhibited higher variation nevertheless with a clear tendency to higher-than-average values at the shallow areas sampled. Populations at shallow localities in the south of the lake also yielded haplotype networks exhibiting longer branches with many missing haplotypes, and mismatch distributions spanning larger genetic distances than populations at deep shorelines (Fig. 4).

Within each species, differences in haplotype diversity, nucleotide diversity and theta were rarely significant between populations of deep and shallow shorelines (Table 4), although diversity values tended to be lower in populations from deep shorelines. Nevertheless, across all species, effect of shoreline type was highly significant for the three analysed genetic diversity indices (Table 5).

**Table 5:** Results of the anova performed across all species, with response variables haplotype diversity (Hd), nucleotide diversity (Pi) and theta, and effects species, shoreline type and interaction between both (sp x sh)

Factor	df	Sum Sq	F	p-value
HD				
Species	2	0.1886	4.767	**0.0185**
Shoreline	1	0.7266	36.726	**0.00000351**
sp × sh	2	0.2118	5.353	**0.0123**
Residuals	23	0.455		
Pi				
Species	2	0.0000489	1.113	0.34555
Shoreline	1	0.0001801	8.207	**0.00876**
sp × sh	2	0.0000262	0.597	0.55903
Residuals	23	0.0005047		
θ				
Species	2	0.0001304	2.26	0.12699
Shoreline	1	0.0003594	12.461	**0.00179**
sp × sh	2	0.0000711	1.232	0.31018
Residuals	23	0.0006634		

df, degrees of freedom.

Bold values denote significant effects.

For each population of each species, estimated neutrality tests and significance of departures from neutrality are shown in Table S3 (Supporting Information). No marked difference was detected between deep and shallow shorelines, with neutrality being rejected for some populations of some species irrespective of the shoreline type. We note that our demographic reconstructions and dating analysis could be affected by the presence of selection on mtDNA genes. However, neutrality tests were for the most part not significant, and there was no association between shoreline type and deviations from neutrality. Therefore, we find it unlikely that our results would be significantly affected even in the presence of slight departures from neutrality in a minority of the populations analysed.

Estimated demographic histories for each species and locality are shown in Figs 7. For *Tropheus* sp., we show only results when using the most abundant mtDNA lineage(s) within each locality. In some of the analyses performed (populations of *E. cyanostictus* and *Tropheus* sp. from locality 10, and population of *V. moorii* from locality 8), convergence was not attained after 100 million generations. In these cases, we simplified the nucleotide substitution model (using a single category of mutations and no invariable sites) and used 5 (instead of 10) groups for the Bayesian skyline, and ran the analyses again. In one case (*E. cyanostictus* population from locality 8), even this simplified model did not reach convergence (low ESS for most parameters), and, we do not present these results. Analyses of the other two populations reached convergence with the simplified model, and results are included in Figs 6 and 7. Populations from deep shorelines across all species exhibited markedly shorter demographic histories, as the lower genetic diversity present in these populations entails shorter times to coalescence of all lineages and thus does not allow us to recover traces of older demographic events. Populations from these deep shorelines also exhibited more stable population sizes, when compared to the demographic histories of populations from shallow localities. However, confidence intervals in all analyses are quite large, and thus, these results should be interpreted with caution.

**Fig. 5:**
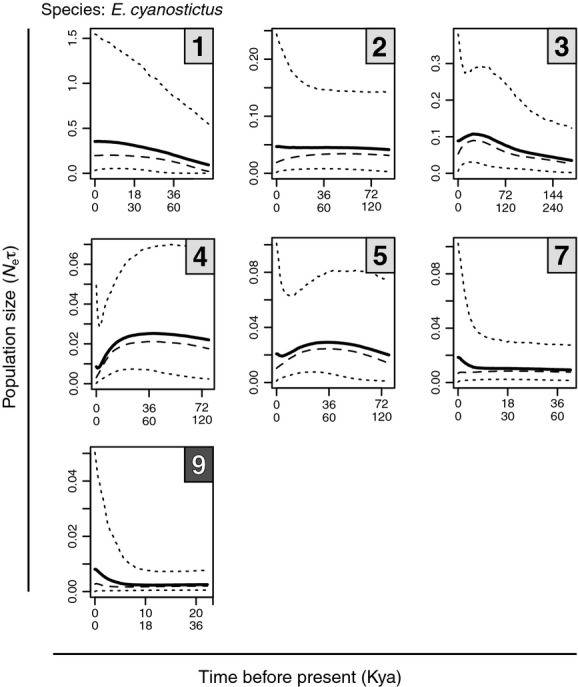
Demographic histories of populations of *E. cyanostictus* reconstructed in the program beast. Numbers inside each graph denote locality of origin. Thick lines represent means, dashed lines medians and dotted lines the 95% confidence distribution of the effective population size (scaled by mutation rate) in each case. On the x-axes, time is given in thousand of years before present (Kya) when using a substitution rate of 0.057 (up) or 0.0325 (down) substitutions per million of years (Sturmbauer *et al*. 2001). Note that the different graphs have different x- and *y*-axis scales.

**Fig. 6:**
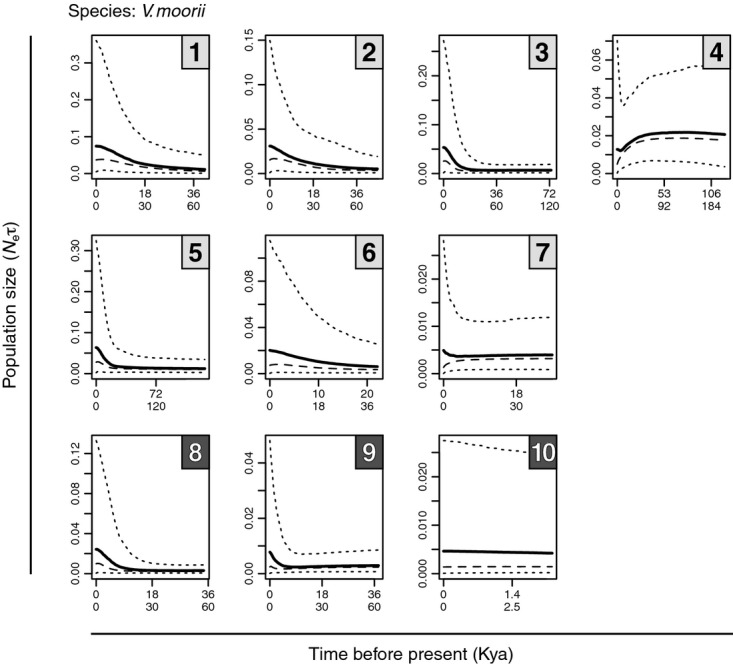
Demographic histories of populations of *V. moorii* reconstructed in the program beast. Data presented as in previous figure (see details in legend to Fig. 5).

**Fig. 7:**
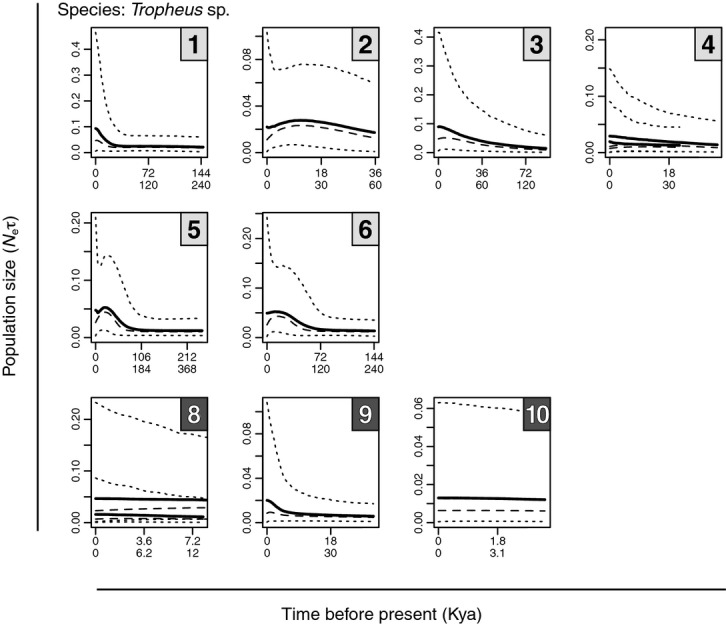
Demographic histories of populations of *Tropheus* sp. reconstructed in the program beast. Data presented as in previous figures (see details in legend to Fig. 5). In localities 4 and 8, we show two demographic reconstructions, corresponding to the two, roughly equally abundant mtDNA lineages found.

## Discussion

We compared patterns of genetic diversity and demographic dynamics of co-distributed cichlid fish species inhabiting both deep and shallow shorelines of Lake Tanganyika. Genetic differentiation between populations of each species was generally high, in accordance with previous studies, where strong geographical structuring has been described for all three species (Sturmbauer & Meyer 1992; Verheyen *et al*. 1996; Rüber *et al*. 1999; Duftner *et al*. 2006; Sefc *et al*. 2007). Thus, conspecific individuals inhabiting different shoreline sections of the lake can be regarded as separate populations, whose evolutionary histories can be addressed separately.

According to our working hypothesis, localities at very deep shorelines of the lake would represent environmental refugia during periods of reduced water level, while populations from the shallow shorelines would repeatedly experience dramatic reductions to their habitat availability and population sizes, followed by re-colonization events seeded by populations from deeper shorelines. This scenario would be in analogy to the several well-studied cases of terrestrial or riverine environmental refugia during glaciations in Europe (e.g. Hewitt 1999, 2000) and would posit that populations from shallow shorelines would be relatively young and genetically less diverse than those inhabiting the deep shorelines.

As expected, we detected a significant association between shoreline type (deep vs. shallow) and measures of genetic diversity within populations (Tables 4 and 5). This association was particularly strong when we compared diversity indexes across species (Table 5), with a highly significant effect of shoreline type (after accounting for species) upon haplotype diversity, nucleotide diversity and theta estimates. Our demographic reconstructions also support our *a priori* expectations: in general, populations inhabiting the shallow areas in the southern end of the lake showed traces of recent population growth, while populations from deep shorelines exhibited more stable demographic histories (Figs 7). This result should be taken with caution not only because the pattern was not always clear (some populations inhabiting deep shorelines also showed traces of recent population growth, while some populations from shallow locations did not), but also due to the limitations of mtDNA markers in recovering the demographic history of populations: the stochastic nature of the coalescent process; and the effect of mtDNA introgression or sex-specific behaviour (which can lead to different evolutionary histories of mtDNA and nuclear markers). Regarding the former effect, two observations suggest that our results reflect, at least to some extent, the true demographic history of the populations analysed: different populations inhabiting the shallow shorelines exhibited very similar population size changes; and our dating for the onset of these population expansions (50–100 Kya) is in agreement with several previous studies in highlighting the effect of late Pleistocene water-level changes in East African lakes (Cohen *et al*. 2007; Scholz *et al*. 2007; Genner *et al*. 2010; Koblmüller *et al*. 2011). Regarding the possibility of different evolutionary histories recovered from mtDNA and nuclear DNA markers, we note that previous studies with *V. moorii* and *E. cyanostictus* recovered highly congruent patterns from both mtDNA and microsatellite data (Duftner *et al*. 2006; Sefc *et al*. 2007).

The observed relationship between shoreline type and genetic diversity was, however, the opposite of our expectation: the genetic diversity estimates for populations inhabiting the deep shorelines were significantly lower than those for populations inhabiting the shallow shorelines at the southern end of the lake. This is surprising, given that populations from the deep areas are likely to be older, and to have had more constant population sizes, and as such should have accumulated and maintained higher levels of diversity at neutral genetic markers.

Our finding is even more surprising as several other studies have indeed reported increased levels of genetic diversity in older or more stable habitats. For instance, Fauvelot *et al*. (2003) compared genetic diversity of coral fish populations inhabiting both lagoon and outer slope habitats. The authors found that the older populations inhabiting the outer slopes, which have experienced comparatively mild changes in habitat availability due to sea level changes, exhibited significantly higher haplotype diversity than the younger populations from lagoons (whose habitat has been dramatically reduced due to Holocene sea level changes). Likewise, Knaepkens *et al*. (2004) observed a positive relationship between population size and genetic diversity in fragmented populations of the European bullhead, while McCusker & Bentzen (2010) reported on a positive correlation between population size and genetic diversity across a variety of freshwater and marine fish species. Concerning East African cichlids, Koblmüller *et al*. (2011) studied populations of *Tropheus* sp. from the southern end of the lake (between localities numbers 4 and 7 in Fig. 1) and found a positive correlation between expected habitat stability (as inferred by shoreline inclination) and measures of genetic diversity.

### Causes for increase in genetic diversity in shallow shorelines

The expected relationship between population age and genetic diversity would suggest that the populations inhabiting the shallow, southern localities are older, having had more time to accumulate genetic diversity in neutral markers. However, an older age for these populations is at odds with the bathymetric profile of the lake, and the known water-level fluctuations in Lake Tanganyika.

An alternative explanation is that these populations have higher effective population sizes than those at deeper shoreline sections. This could be due to locations at shallower shorelines exhibiting a gentler slope, leading to wider bands of appropriate habitat in these locations. However, the actual slope varies in both areas: we find big rocks, boulders and cobble shores in various inclinations in both the deep and the shallow shorelines. Furthermore, the phylopatric nature of all three species analysed means that they form populations isolated by distance even over continuously rocky habitat (see e.g. Duftner *et al*. 2006 for *V. moorii*; and Sefc *et al*. 2007 *for T. moorii* and *E. cyanostictus*). Thus, higher habitat availability would likely result in more populations (isolated by distance) instead of higher effective population sizes of each population.

The high genetic diversity observed in the shallow localities could also be the result of higher habitat heterogeneity: these shorelines could accumulate more sediment than deep shoreline locations, resulting in more important barriers to gene flow for the rock-dwelling species analysed in this study. However, as outlined above, both shoreline inclination and habitat heterogeneity vary in both classes of locations. Thus, habitat heterogeneity is not always higher in shallow locations and in itself is unlikely to explain the observed differences between shallow and deep shorelines.

Finally, the higher than expected genetic diversity observed in the shallow localities could be an unexpected result of the frequent water-level fluctuations in Lake Tanganyika. While these populations are necessarily younger than those located at deep shorelines, the fluctuations in water level may have resulted in periodic strong episodes of migration between otherwise isolated populations inhabiting the shallow shorelines. Genetic diversity arising in each of these populations could thus spread to other populations due to these environmental forces, leading to the establishment of a certain type of metapopulation dynamics (e.g. Hastings & Harrison 1994), effectively enhancing the number of mtDNA haplotypes across the shallow shorelines via frequent admixes–dispersal events. Under such a scenario, theoretical work suggests that higher rates of migration between demes or higher extinction and recolonization rates within demes would result in high levels of genetic diversity (Wakeley & Aliacar 2001). Furthermore, the ‘rescue effect’ sensu Brown & Kodric-Brown (1977), if applied to genetic variation instead of species diversity, can explain the maintenance of a higher number of different haplotypes within each population (e.g. Ingvarsson 2001). Under this scenario, haplotypes that go extinct in one or more demes might be re-introduced to these demes by the periodic reshuffling process brought about by lake-level fluctuations.

### Causes for decrease in genetic diversity in deep shorelines

A first possible explanation for the reduced genetic diversity of populations inhabiting deep shorelines would be that these shorelines were only more recently colonized than the shallow ones. However, we cannot see any reason why these deeper areas should have been colonized later than the southern, shallower shorelines. In fact, the latter shorelines were certainly completely dry several times since 500 Kya (Lezzar *et al*. 1996; Cohen *et al*. 1997), while the deeper areas are likely to have been more suitable to sustain rock-dwelling cichlid species for much longer periods of time. We cannot completely exclude the alternative hypothesis that shores in the deeper areas also become unsuitable during periods of low lake level for the three species analysed, because we do not have data on the putative shoreline composition at several hundred metres below current levels. Nevertheless, the shores in these deep areas are very steep and most often drop continuously to c. 1400 m below current surface level, so that they are likely to be composed of rocky substrate with very little sandy areas, as the inclination itself prevents the deposition of sand on a large scale. Therefore, it seems likely that the substrate of the deep shorelines at lowered lake level would be suitable for the studied rock-dwelling species.

A second possible explanation is that the deep localities covered in this study (localities 8–10 in Fig. 1) would have experienced specific environmental conditions, and would therefore not be representative for deep shorelines in general. For instance, they could have experienced increased human or geologically induced habitat disturbance that would have made them unsuitable habitats until very recently. However, our own unpublished data suggest that populations of closely related species (*Tanganicodus irsacae* and *Tropheus brichardi*) inhabiting different deep shorelines at the central sub-basin of Lake Tanganyika exhibit similarly reduced levels of genetic diversity, thus suggesting that the pattern we recovered is representative for populations inhabiting deep shorelines throughout the lake.

As a third hypothesis, the species analysed could have only recently originated at the southern end of the lake and subsequently expanded their distribution range towards the deeper regions at the central region of the southern sub-basin. This explanation is at odds with several lines of evidence from phylogeographic studies of *Tropheus* sp., whose lake-wide distribution has been connected to the rise of the lake level starting 1.1 Mya (Baric *et al*. 2003; Sturmbauer *et al*. 2005), or the inferred old age of *V. moorii*, one of the oldest members of the Lamprologini tribe and thought to have originated >1 Mya (Sturmbauer *et al*. 1994). Members of the Eretmodini tribe have also most likely inhabited the central regions of the southern sub-basin during major water-level low stands, as revealed by the sharing of haplotypes between populations from opposite sides of the lake (Verheyen *et al*. 1996; Rüber *et al*. 1999). Thus, the combined existing evidence rules out that these species are of recent origin, and it is therefore unlikely that the pattern of reduced genetic diversity could be explained by an allegedly recent origin of these three species on the southern end of the lake and their subsequent expansion towards the deeper shorelines of the southern sub-basin.

Overall, it seems unlikely that our results could be explained by a recent origin of the populations inhabiting the deep shorelines analysed in this work. Instead, they seem to reflect a real biological mechanism that must explain the decrease in genetic diversity in the older and more stable populations analysed in this work. The most likely explanation seems to be that in the deep shorelines, the connectivity between populations is not strongly affected by water-level fluctuations, so that the metapopulation dynamics suspected to have occurred in the shallow areas are absent from deep localities. This scenario entails that rare haplotypes have a greater chance to go extinct via lineage sorting in populations inhabiting deep shorelines, because once they do go extinct, they are not replaced by fusion with other populations, resulting in only the most abundant haplotypes remaining in the populations for longer periods.

It should be noted that the reduction in haplotype diversity observed in populations from deep shorelines is surprisingly large. For instance, in locality 9, only 4 haplotypes were found in *E. cyanostictus* (37 individuals collected) and *V. moorii* (48 specimens analysed). It is thus possible that other processes in addition to lineage sorting have reduced the variation of these populations even further. One might argue that relatively frequent selective sweeps in these areas could lead to strong reduction in haplotype diversity due to the selective advantage of the sweeping haplotype. Indeed, a similar explanation has been advanced to cope with the observation that, across a variety of taxa, species exhibiting larger effective population sizes do not exhibit comparably higher mtDNA genetic diversity (Bazin *et al*. 2006). Under this scenario, while larger population sizes would entail a faster pace of generation of new haplotypes, they would also cause an increased number of new, potentially selectively advantageous mutations. This would increase the frequency of selective sweeps, which would periodically erase genetic diversity in these populations (‘genetic draft’ cf. Gillespie 2000; Bazin *et al*. 2006). While we do not have any direct evidence for a role for selection in our results, it should be noted that the reduction in genetic diversity estimates observed in the deep shorelines’ populations is higher for *E. cyanostictus* and *V. moorii* than for *Tropheus* sp. (Table 3 and Figs 4). This observation lends some support to the ‘genetic draft’ hypothesis: *E. cyanostictus* and *V. moorii* are monogamous breeders, while *Tropheus* sp. are polygamous (Kohda *et al*. 1997; Yamaoka *et al*. 1997; Yuma & Kondo 1997). Thus, for the same census size, the two former species would be expected to exhibit higher effective population sizes and under this hypothesis result in more reduced genetic diversity estimates.

### Water-level fluctuations, metapopulation dynamics and genetic diversity in the East African cichlid fauna

Arnegard *et al*. (1999) was the first to propose a role for metapopulation dynamics to explain the rapid evolution in East African cichlids. These authors combined detailed bathymetric data, historical observations and genetic data of a rock-dwelling Malawian cichlid to detect traces of repeated episodes of isolation and secondary contact among cichlid populations caused by lake-level changes in Lake Malawi. They hypothesized that lake-level changes would forcibly move cichlid populations between isolated rocky outcrops, thus increasing levels of gene flow between initially distant populations. Our study represents the first independent study that seems to support Arnegard *et al*. (1999) hypothesis and highlights the potential role of metapopulation dynamics in explaining the rapid pace of evolution of the East African cichlid faunas. Metapopulation dynamics may affect the evolution and speciation of cichlid fish in at least two ways. First, drift may operate independently on the genes responsible for mate choice on isolated rocky patches, leading to increased speciation rates as populations in isolated rocky outcrops evolve pre- or postzygotic isolation mechanisms (Arnegard *et al*. 1999). Second, the higher amount of genetic variation maintained across populations can fuel local adaptation of populations (Williams 1966), as well as represent a source of standing genetic variation which could allow populations to quickly respond to changing environmental conditions (e.g. Barrett & Schluter 2008). In view of the many well-documented cases of hybridization and introgression in East African cichlids and their evolutionary importance (e.g. Seehausen 2004), exchange of locally adapted genes among previously isolated populations during secondary contact (as caused by water-level fluctuations) could also lead to faster adaptation of populations to new habitats or changing environmental conditions. Finally, given that similar shallow shorelines exist in the other East African Great Lakes, it seems plausible that our results for Tanganyika cichlids may equally apply to the very high diversification and speciation rates reported for the cichlid species flocks in these lakes. In this context, it is interesting to note that Lake Victoria (the youngest among these lakes) is characterized by the absence of deep shorelines while exhibiting the highest speciation rates for East African cichlids (Verheyen *et al*. 2003).

## Data accessibility

DNA sequences: GenBank Accession numbers detailed in Table S1, Supporting Information. Sequence alignments: doi:10.5061/dryad.m2661.

## Supporting information

**Table S1** Detailed ID of individuals analysed (including accession numbers).

**Table S2** PCR protocols.

**Table S3** Result of neutrality tests.
