# Supplementary material for: Water-level fluctuations and metapopulation dynamics as drivers of genetic diversity in populations of three Tanganyikan cichlid fish species
Source: Mol Ecol. 2013 Jul 10;22(15):3933–48. doi: 10.1111/mec.12374 (PMC3763204; doi:10.1111/mec.12374)
Supplement: Supplementary file 2 [file mec0022-3933-SD2.doc]

Supplementary material Table S2: PCR protocols and primers used for each species

| Gene | PCR Mix | PCR cycle | Primers |
| --- | --- | --- | --- |
| Control region  *E. cyanostictus* | DNTPs : 0,2 mM  Sigma RedTaq: 1 unit/reaction  10x RedTaq buffer: 1 unit/reaction  primer: 0,2 µM each  dna: 100-200 nmol | 94° : 3’  94° : 1’  56° : 1’  72° : 1’30’’  72° : 10’  30 cycles | LPRO2: 5'-CTCTCACCCCTAGCTCCCAAAG-3'  TDK-DH4-SHORT: 5'-GATCCCATCTTCAGTGTTATGC-3' |
| Control region  *T. moorii* | DNA: 5-10 ng  dNTPs : 0.05 mM  Biotherm Taq Polmerase: 1 unit  10 x Taq Polymerase buffer: 1x  Primer: 0.25 µM each  BSA: 0.4 µg/µl | 94° : 3’  94° : 30’’  52° : 30’’  72° : 1’  72° : 10’  40 cycles | L15926T: 5’-cagcgccagagcgccggtcttg-3’  TDKDH4T: 5’-tccgtcttaacatcttcagtgttatgc-3’ |
| Control region  *V. moorii* | DNA: 5-10 ng  dNTPs : 0.05 mM  Biotherm Taq Polmerase: 1 unit  10 x Taq Polymerase buffer: 1x  Primer: 0.25 µM each  BSA: 0.4 µg/µl | 94° : 3’  94° : 30’’  52° : 30’’  72° : 1’  72° : 10’  40 cycles | L15926T: 5’-cagcgccagagcgccggtcttg-3’  TDK-DH4-short: 5’-tccgtcttaacatcttcagtgttatgc-3’ |

Primers ‘LPRO2’ and ‘TDK-DH4-short’ were published by Nevado et al. (2009) Molecular Ecology 18, 4240-4255.

L15926T modified from Kocher et al. (1989) Proc. Natl. Acad. Sci. USA 86:6196-6200.
