# Supplementary material for: Water-level fluctuations and metapopulation dynamics as drivers of genetic diversity in populations of three Tanganyikan cichlid fish species
Source: Mol Ecol. 2013 Jul 10;22(15):3933–48. doi: 10.1111/mec.12374 (PMC3763204; doi:10.1111/mec.12374)
Supplement: Supplementary file 3 [file mec0022-3933-SD3.doc]

Supplementary material Table S3: Estimated genetic diversity statistics (N- number of specimens; S- number of segregating sites; h- number of haplotypes) and neutrality indexes (T D- Tajima’s D; FL D – Fu and Li’s D; FL F- Fu and Li’s F; Fs- Fu’s Fs; R2- Ramos-Onsins R2 test) calculated for the different populations of each species using the control region. For neutrality tests, we report both the value of the statistic and the significance of its departure from neutrality inside parenthesis (significant departures marked in bold). For *T. moorii*, when more than 1 lineage was found within the same locality, we provide these statistics for all specimens from that locality, and as well for only the specimens from the most abundant lineage(s).

1. *V. moorii*

| Loc Nr | 1 | 2 | 3 | 4 | 5 | 6 | 7 | 8 | 9 | 10 |
| --- | --- | --- | --- | --- | --- | --- | --- | --- | --- | --- |
| N | 41 | 42 | 46 | 41 | 35 | 24 | 37 | 50 | 48 | 12 |
| S | 20 | 17 | 15 | 21 | 22 | 7 | 8 | 10 | 9 | 3 |
| h | 19 | 13 | 12 | 13 | 15 | 7 | 6 | 7 | 4 | 2 |
| T D | -1,13 (0,11) | -1,30 (0,09) | -1,30 (0,10) | -1,04 (0,16) | 0,03 (0,56) | 0,12 (0,56) | 0,02 (0,56) | -1,22 (0,12) | 1,90 (0,96) | **-1,63 (0,02)** |
| FL D | -1,12 (0,15) | -0,78 (0,19) | -0,98 (0,19) | -0,38 (0,30) | -0,46 (0,29) | 0,14 (0,55) | 1,31 (0,94) | -0,86 (0,19) | 0,7 (0,78) | **-1,95 (0,01)** |
| FL F | -1,33 (0,12) | -1,12 (0,17) | -1,28 (0,13) | -0,71 (0,25) | -0,35 (0,36) | 0,16 (0,55) | 1,07 (0,86) | -1,15 (0,15) | 1,28 (0,92) | **-2,12 (0,01)** |
| Fs | **-9,89 (0,0)** | **-4,33 (0,03)** | **-3,60 (0,05)** | -1,76 (0,25) | -2,21 (0,20) | -0,52 (0,41) | 0,60 (0,64) | -0,78 (0,37) | 6,12 (0,97) | 1,05 (0,79) |
| R2 | 0,07 (0,10) | 0,07 (0,07) | 0,07 (0,11) | 0,09 (0,31) | 0,11 (0,55) | 0,15 (0,71) | 0,12 (0,54) | 0,07 (0,16) | 0,17 (0,98) | 0,28 (0,81) |

B) *E. cyanostictus*

| Loc Nr | 1 | 2 | 3 | 4 | 5 | 6 | 7 | 8 | 9 | 10 |
| --- | --- | --- | --- | --- | --- | --- | --- | --- | --- | --- |
| N | 38 | 11 | 40 | 38 | 28 |  | 29 | 17 | 37 |  |
| S | 25 | 15 | 44 | 15 | 20 |  | 15 | 1 | 7 |  |
| h | 25 | 8 | 27 | 11 | 14 |  | 9 | 2 | 4 |  |
| T D | **-1,52 (0,04)** | 0,10 (0,57) | -1,20 (0,11) | 0,47 (0,73) | **-0,30 (0,4)** |  | -1,08 (0,14) | -1,16 (0,09) | 1,76 (0,96) |  |
| FL D | **-2,79 (0,01)** | 0,44 (0,66) | -1,77 (0,06) | 0,28 (0,61) | -0,51 (0,28) |  | **-2,35 (0,03)** | -1,48 (0,09) | 0,53 (0,7) |  |
| FL F | **-2,80 (0,01)** | 0,40 (0,63) | **-1,86 (0,05)** | 0,40 (0,68) | -0,52 (0,32) |  | **-2,29 (0,04)** | -1,59 (0,08) | 1,07 (0,86) |  |
| Fs | **-20,96 (0,0)** | -1,34 (0,22) | **-12,59 (0,00)** | -0,38 (0,48) | -3,12 (0,09) |  | -1,27 (0,24) | -0,75 (0,31) | 4,17 (0,96) |  |
| R2 | **0,06 (0,02)** | 0,16 (0,50) | 0,07 (0,07) | 0,13 (0,73) | 0,11 (0,42) |  | 0,09 (0,20) | 0,24 (0,48) | 0,19 (0,97) |  |

*c) Tropheus* sp. *moorii*

| Loc Nr | 1 | 2 | 3 | 4 | 5 | 6 | 7 | 8 | 9 | 10 |
| --- | --- | --- | --- | --- | --- | --- | --- | --- | --- | --- |
| N | 55  35 | 46 | 26 | 50  23  19 | 46  42 | 46 |  | 40  21  19 | 48 | 25  15 |
| S | 36  23 | 17 | 24 | 38  10  12 | 41  31 | 35 |  | 37  23  6 | 12 | 28  4 |
| h | 28  19 | 16 | 17 | 19  7  8 | 25  22 | 21 |  | 19  12  7 | 12 | 12  4 |
| T D | 0,36 (0,70)  -0,07 (0,55) | -1,38 (0,07) | -1,13 (0,12) | 1,54 (0,95)  -1,25 (0,10)  -1,35 (0,08) | -0,87 (0,22)  -0,92 (0,18) | -0,72 (0,23) |  | 0,83 (0,84)  -1,31 (0,09)  -0,66 (0,29) | -0,35 (0,39) | 0,039 (0,56)  **-1,52 (0,02)** |
| FL D | -0,58 (0,24)  1,03 (0,17) | -0,84 (0,19) | -1,89 (0,06) | -0,66 (0,79)  -0,08 (0,44)  -1,76 (0,07) | 0,11 (0,51)  -0,89 (0,19) | -1,03 (0,15) |  | 0,03 (0,48)  -1,03 (0,17)  -0,13 (0,42) | -0,004 (0,45) | -0,50 (0,25)  -1,47 (0,07) |
| FL F | -0,28 (0,40)  -0,84 (0,21) | -1,21 (0,13) | **-1,94 (0,05)** | 1,16 (0,91)  -0,5 (0,33)  -1,90 (0,07) | -0,29 (0,40)  -1,06 (0,15) | -1,10 (0,16) |  | 0,37 (0,66)  -1,30 (0,13)  -0,32 (0,36) | -0,14 (0,43) | -0,39 (0,33)  -1,69 (0,06) |
| Fs | -6,04 (0,06)  **-5,19 (0,03)** | -8,14 (**0,00**) | **-7,90 (0,0)** | 1,22 (0,71)  -1,41 (0,19)  -2,06 (0,1) | **-7,82 (0,03)**  **-8,09 (0,01**) | -4,41 (0,09) |  | -0,68 (0,42)  -3,20 (0,07)  **-2,53 (0,05)** | -2,46 (0,14) | -0,10 (0,51)  -1,29 (0,09) |
| R2 | 0,12 (0,76)  0,12 (0,58) | 0,06 (0,06) | 0,07 (0,05) | 0,17 (0,99)  0,09 (0,05)  0,10 (0,13) | 0,08 (0,19)  0,08 (0,18) | 0,09 (0,27) |  | 0,14 (0,87)  0,09 (0,07)  0,11 (0,14) | 0,10 (0,52) | 0,12 (0,51)  0,14 (0,17) |
